# Evaluating teachers’ knowledge and attitude toward food allergy and anaphylaxis: a pilot simulation-based questionnaire study in Rabigh elementary schools

**DOI:** 10.3389/fpubh.2025.1631540

**Published:** 2025-10-29

**Authors:** Nojoud Faqerah, Saddiq B. Habiballah, Majdi Damanhori, Abdullah Alsaggaf, Manal Ahmed Halwani

**Affiliations:** ^1^Department of Medical Microbiology, Faculty of Medicine, King Abdulaziz University, Rabigh, Saudi Arabia; ^2^Department of Pediatrics, Faculty of Medicine, King Abdulaziz University, Jeddah, Saudi Arabia; ^3^Department of Human Anatomy, Faculty of Medicine, King Abdulaziz University, Rabigh, Saudi Arabia; ^4^Unit of Allergy and Immunology, Department of Pediatrics, Faculty of Medicine, King Abdulaziz University, Jeddah, Saudi Arabia; ^5^Department of Emergency Medicine, Faculty of Medicine, King Abdulaziz University, Jeddah, Saudi Arabia

**Keywords:** pre–post simulation, simulation-based learning, teacher knowledge, food allergy, anaphylaxis

## Abstract

**Background:**

Simulation-based learning is an effective tool for educating teachers and school staff about anaphylaxis. This pilot exploratory study aimed to assess knowledge and attitudes toward food allergy and anaphylaxis policies and protocols in schools for the management of severe allergic reactions.

**Materials and methods:**

A simulation-based education program was conducted using a standardized case scenario in elementary schools in Rabigh, targeting teachers and staff from randomly selected schools. Knowledge and attitudes were assessed before and after the simulation using structured questionnaires. Paired pre–post responses (*n* = 97) were analyzed. McNemar’s test was applied for categorical outcomes, and the Wilcoxon signed-rank test was used for ordinal attitude scores. Data were summarized as frequencies and percentages, with a significance level set at *p* < 0.05.

**Results:**

A total of 101 teachers and staff from six elementary schools participated, with 97 completing both pre- and post-simulation assessments. Recognition of key anaphylaxis symptoms improved significantly post-intervention, particularly for swelling of the lips, tongue, face, and eyes (55.4% vs. 79.4%) and shortness of breath (28.7% vs. 78.4%) (both *p* < 0.001, McNemar’s test). Attitudes toward anaphylaxis management protocols also improved, with a significant reduction in median attitude scores post-simulation (*p* = 0.001, Wilcoxon signed-rank test). Readiness to use an epinephrine auto-injector increased markedly from 18.8% before the intervention to 84.5% after the intervention (*p* < 0.001).

**Conclusion:**

This pilot exploratory study demonstrates that simulation-based education can significantly improve teachers’ knowledge, attitudes, and readiness to manage food allergy and anaphylaxis. While the findings are promising, they should be interpreted with caution due to the modest sample size and single-city setting. Larger, multi-center studies.

## 1 Introduction

The prevalence of food allergies varies globally, with estimates ranging from 8 to 10% in developed countries (1). In Saudi Arabia, the prevalence averaged around 15.2%, with significant regional variability (2). Anaphylaxis is an immediate, life-threatening, and immune-mediated reaction to an allergic trigger (3). As such, prompt recognition and treatment are required to prevent severe outcomes, highlighting the importance of awareness and education regarding food allergies.

A study from Spain found that 10–18% of food-related allergic reactions occurred while children were at school, highlighting the importance of school readiness for such events (4). A school nurse’s presence differs from the standard practice in Saudi Arabia; however, recently, appointing a teacher as a health liaison has become mandatory for all schools at all educational stages. Multiple studies have highlighted the critical deficiency in training and resources available to the school’s staff, who have daily contact with children at risk of experiencing an allergic reaction, emphasizing the need for comprehensive training programs to equip them with the necessary skills to manage allergic reactions effectively (4). A study from Saudi Arabia raised a major red flag that most school staff in one region lacked the simple and basic knowledge of food allergy, recognizing symptoms, and managing anaphylaxis (5). One essential area where school staff demonstrated a significant lack of knowledge and skills was recognizing the symptoms and signs of severe food-induced allergic reactions, as well as when and how to use adrenaline auto-injectors (4, 5). This gap in training not only puts students at risk but also creates a sense of anxiety among parents, who may feel their children’s safety is compromised while at school.

Most of the previous studies showed the level of awareness of anaphylaxis in Saudi Arabia, but they failed to provide a real solution. Simulation is an excellent educational tool, enabling educators to expose trainees to real-world scenarios without compromising patient safety (6). This tool has shown great success in medical education for healthcare practitioners (6). Studies have demonstrated that case simulations are more effective than standard lectures for emergency situational training (7–9). It can be extrapolated for use by school staff as necessary. In this study, we utilized simulation-based training to evaluate the knowledge and skills of school staff in recognizing the symptoms and signs of anaphylaxis and its management before and after simulation. We also assess attitudes toward guidelines and protocols in schools for managing severe allergic reactions. This approach is unique in Saudi Arabian schools, where food allergy prevalence is rising and trained medical staff are often unavailable. By offering a practical and scalable educational tool, simulation-based training addresses an urgent public health gap. This pilot study provides preliminary evidence of its feasibility and impact in Saudi Arabian schools, while laying the groundwork for larger-scale investigations.

## 2 Materials and methods

### 2.1 Participants

This study utilized a simulation-based education approach, employing a case scenario conducted in six randomly selected elementary schools in Rabigh. A quasi-experimental pre–post intervention design was applied to evaluate changes in knowledge and attitudes toward food allergy and anaphylaxis among schoolteachers and staff. Both male and female participants were eligible for participation. The sample size was calculated using the single-proportion formula: *n* = (Z^2^_{*α*/2} · p(1 − p)) / d^2^, where p represents the estimated prevalence of good awareness among schoolteachers (0.145), d is the margin of error (0.07), and Z is the standard normal deviate corresponding to a 95% confidence level (1.96). Substituting these values yielded a minimum required sample size of approximately 97 participants. To accommodate a potential 20% non-response or attrition rate, the final target sample was adjusted to 121 participants. Ultimately, 101 participants completed the pre-test, and 97 completed the post-test.

Data collection took place between February and April 2024, during the academic term when teachers and staff were available for scheduled simulation sessions. The inclusion criteria encompassed all mainstream elementary schools within Rabigh city and surrounding villages, while special education schools were excluded.

### 2.2 Questionnaire design

Pre- and post-simulation knowledge and attitude evaluations were conducted using pre- and post-questionnaires (10, 11). To align with the local population and study aims, modifications included simplification of wording, the inclusion of contextual examples relevant to school settings, and translation into Arabic with subsequent back-translation into English to ensure semantic equivalence. A pilot test involving 10 teachers was conducted to confirm clarity and feasibility. Internal consistency was acceptable (*Cronbach’s α > 0.70*).

The questionnaire covered three domains: (a) the importance of having guidelines and protocols for managing severe allergic reactions in schools; (b) recognition of anaphylaxis signs and symptoms; and (c) immediate management and response.

### 2.3 Simulation

The hybrid simulation program combined two professionally developed video scenarios with a task trainer. Each session lasted approximately 90 min, was conducted in groups of 10–12 participants, and was facilitated by pediatric emergency physicians experienced in the management of allergy and anaphylaxis. Scenarios depicted a student developing anaphylaxis (e.g., cough, wheeze, hypoxia) followed by appropriate management steps. All teachers and staff actively participated in scenario discussions and case resolution. Practical training included the use of a Jext® trainer (ALK-Abelló, UK), a needle-free, reusable device that mimics the design and activation steps of an epinephrine auto-injector, enabling participants to practice correct administration technique safely. Each session concluded with a structured debriefing to reinforce best practices and address participant questions.

### 2.4 Statistical analysis

Sociodemographic characteristics and questionnaire responses were summarized using descriptive statistics. Continuous variables were reported as means with standard deviations (SD) or medians with interquartile ranges (IQR), depending on data distribution (assessed via Shapiro–Wilk). Categorical variables were presented as frequencies and percentages.

For inferential analysis, McNemar’s test was used for paired categorical outcomes (e.g., recognition of anaphylaxis symptoms, readiness to use an epinephrine auto-injector). The Wilcoxon signed-rank test was used to compare pre- and post–knowledge and attitude scores, and the Mann–Whitney U test evaluated gender-based differences. Effect sizes were calculated as rank-biserial correlation (Wilcoxon) and Cohen’s r (Mann–Whitney). A *post hoc* power analysis using G*Power 3.1.9.7 indicated that, for a two-tailed Wilcoxon signed-rank test with a medium effect (*r* = 0.4) and *α* = 0.05, the achieved power was 0.84. Analyses were performed using Minitab 22 and IBM SPSS 26. A two-sided *p* < 0.05 was considered statistically significant.

Given the exploratory nature of this educational intervention, no formal adjustments for multiple testing were applied. This is consistent with methodological recommendations that early-phase or pilot studies prioritize detection of potential signals of change over strict family-wise error control; nonetheless, we acknowledge an increased risk of Type I error and interpret secondary findings cautiously (12, 13). Given the modest sample size and single-site design, the study was framed as a pilot exploratory investigation. Attitudes were measured on a 5-point Likert scale (1 = Strongly Agree to 5 = Strongly Disagree), yielding total scores from 10 to 50, with lower scores indicating more favorable attitudes.

### 2.5 Ethical approval

The Research Ethics Committee at King Abdulaziz University, Unit of Biomedical Ethics, with reference number 240–25, recommended granting permission to conduct the project on 22 October 2024. The Research Ethics Committee (REC) is based on the Good Clinical Practice (GCP) Guidelines. We conducted this investigation in accordance with the ethical criteria outlined in the Declaration of Helsinki. All participants received an information sheet and provided written informed consent before participation.

## 3 Results

### 3.1 Participant characteristics

A total of 101 participants completed the pre-test questionnaire. However, 4 participants did not complete the post-test due to reasons such as loss to follow-up or non-response, resulting in a total of 97 participants included in the post-test analysis. Of the total sample, 37 (36.63%) were male and 64 (63.36%) were female. Participants were categorized into six age groups, with the majority falling between 35 and 44 years (*n* = 60; 60.00%) and 45–54 years (*n* = 29; 29.00%). Most participants were teachers (*n* = 80; 79.20%), while 21 (20.80%) were administrative staff. Among 68 participants who specified their teaching subjects, the most common were Arabic (*n* = 12; 17.65%), science (*n* = 10; 14.71%), mathematics (*n* = 9; 13.24%), and Islamic studies (*n* = 9; 13.24%). Regarding previous allergy training, only 21 participants (20.8%) had previously used an epinephrine auto-injector, while 80 (79.2%) had not. General knowledge of allergies was reported by 63 participants (61.17%), with reading (46.27%) cited as the most frequent source, followed by family (25.37%) and friends (14.93%). Only 21 participants (20.8%) had ever used an epinephrine auto-injector, while 80 (79.2%) had not (Table 1).

**Table 1:** Participant characteristics.

Characteristic	*N* (%)
Gender	
Male	37 (36.63)
Female	64 (63.36)
Job	
Teacher	80 (79.20)
Administrator	21 (20.80)
Age (years)	
25–34	10 (10.00)
35–44	60 (60.00)
45–54	29 (29.00)
55–64	1 (1.00)
Missing Age	1
Subjects taught	
Arabic	12 (17.65)
English	8 (11.76)
Islamic studies	9 (13.24)
Mathematics	9 (13.24)
Science	10 (14.71)
Social studies	7 (10.29)
Art	3 (4.41)
PE	3 (4.41)
Other	3 (4.41)
Previous courses related to allergy	
Yes	21 (20.79)
No	80 (79.20)

### 3.2 Identification of anaphylaxis symptoms

**Figure 1:**
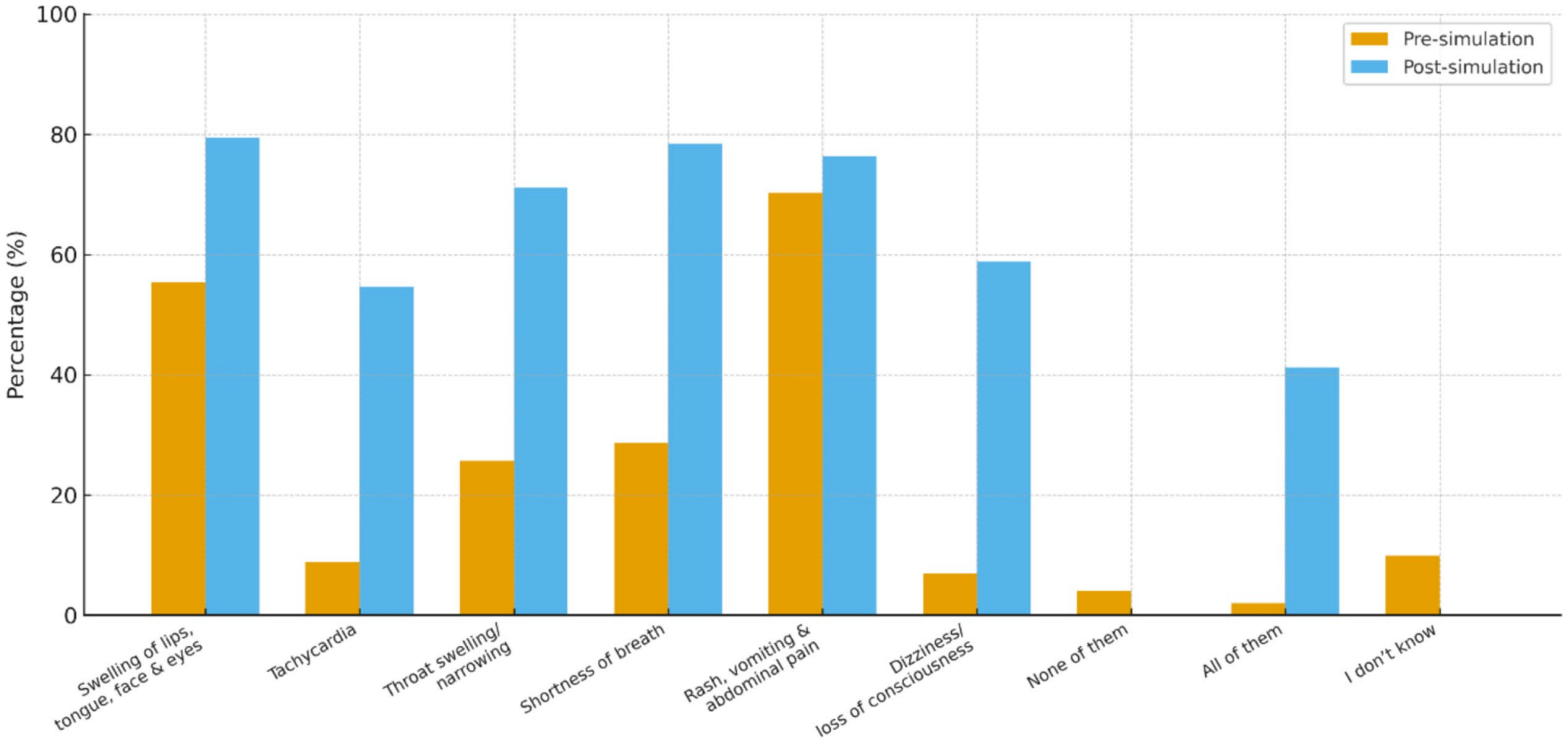
Comparison of recognised allergy symptoms before and after simulation. This figure shows the percentage of participants identifying anaphylaxis symptoms pre- and post-simulation. Recognition increased significantly for most symptoms, with notable reductions in the “None of them” and “I don’t know” categories (all *p* < 0.05, McNemar’s test).

Simulation-based training significantly improved participants’ ability to recognize anaphylaxis symptoms. Table 2 and Figure 1, recognition of key signs such as swelling of the lips, tongue, face, and eyes increased significantly post-intervention (*p* < 0.001; McNemar’s test). Similar improvements were observed for tachycardia, throat swelling, and shortness of breath, and dizziness, all of which showed highly significant gains (*p* < 0.001). The proportion of participants selecting “I do not know” decreased significantly from 9.9 to 0.0% (*p* = 0.002). In comparison, those selecting “None of them” also declined from 4.0 to 0.0%, although this change was not statistically significant (*p* = 0.13) (Table 2). Overall, these findings suggest that the intervention significantly improved symptom recognition across the cohort.

**Table 2:** Recognition of anaphylaxis symptoms before and after the simulation (*n* = 97 paired responses).

Symptoms	Pre (*n*, %)	Post (*n*, %)	McNemar (*p*-value)
Swelling of lips, tongue, face, and eyes	56 (55.4)	77 (79.4)	<0.001
Tachycardia	9 (8.9)	53 (54.6)	<0.001
Swelling and narrowing of throat	26 (25.7)	69 (71.1)	<0.001
Shortness of breath	29 (28.7)	76 (78.4)	<0.001
Rash, vomiting, and abdominal pain	71 (70.3)	74 (76.3)	0.32
Dizziness or loss of consciousness	7 (6.9)	57 (58.8)	<0.001
None of them	4 (4.0)	0 (0.0)	0.13
All of them	2 (2.0)	40 (41.2)	<0.001
I do not know	10 (9.9)	0 (0.0)	0.002

Percentages are based on *n* = 101 pre-simulation and *n* = 97 post-simulation. McNemar’s test used for paired categorical data; *p* < 0.05 considered statistically significant.

### 3.3 Attitudes toward anaphylaxis protocols in schools

There was a statistically significant improvement in attitudes toward school-based anaphylaxis management following the simulation. The Wilcoxon signed-rank test revealed a reduction in attitude scores from a median of 12 (IQR: 10–17) to 10 (IQR: 10–13) post-intervention (*p* = 0.001), with a corresponding rank-biserial correlation of 0.58, indicating a large effect size.

In detail, participants expressed stronger agreement on the importance of implementing measures for allergy preparedness and management after the simulation. The proportion of participants who “strongly agree” on key aspects, such as facilitating direct communication with emergency services (76.2% pre vs. 93.0% post) and defining staff roles in handling emergencies (72.3% pre vs. 80.2% post), increased markedly. The agreement on the need for special supervision during mealtimes (63.4% pre vs. 74.3% post) and rules banning food sharing (61.4% pre vs. 80.2% post) also showed significant improvement. Measures such as banning nuts in schools (58.4% pre vs. 80.2% post) and providing supervision on school buses (58.4% pre vs. 76.2% post) showed notable shifts toward stronger support (Supplementary Table S1). Self-assessed knowledge on a 10-point scale also improved significantly from 4.46 ± 2.38 to 8.21 ± 2.18 (*p* < 0.001; 95% CI [−5, −3]), indicating a statistically significant improvement in knowledge scores following the intervention.

A gender-based analysis revealed a significant pre-intervention difference in attitude scores, with males having a higher mean rank (63.74) than females (44.24; *p* = 0.001; *r* = 0.33, moderate effect). However, post-intervention, the gender difference was no longer statistically significant (*p* = 0.104; *r* = 0.16), suggesting the training had an equalizing effect across genders (Table 3). This indicates that the simulation intervention resulted in similar improvements in attitude across both genders, as no significant difference was observed in post-intervention scores.

**Table 3:** Gender-based comparison of attitudes toward anaphylaxis guidelines and protocols.

Simulation score	Gender	*n*	Mean Rank	*p*-value	Effect size (*r*)
Pre-simulation	Male	37	63.74	0.001*	0.33
	Female	64	44.24		
	Total	101			
Post-simulation	Male	31	54.77	0.104	0.16
	Female	66	46.29		
	Total	97			

Mann–Whitney *U* test; *p* < 0.05 indicates statistical significance (*).

### 3.4 Readiness to use an epinephrine auto-injector

Participants’ readiness to use an epinephrine auto-injector improved significantly after the simulation. The proportion of participants who reported being ready increased from 18.8% before the simulation to 84.5% after the simulation, while those who were unprepared decreased from 30.7 to 4.0%. The proportion of unsure participants declined from 50.5 to 11.5%. These changes were statistically significant (*p* < 0.001; McNemar’s test), demonstrating that the simulation substantially enhanced both confidence and preparedness to use an epinephrine auto-injector (Table 4).

**Table 4:** Readiness to use an epinephrine auto-injector (*n* = 97 paired responses).

Question	Response	Pre-simulation (*n*, %)	Post-simulation (*n*, %)	McNemar *p*-value
Do you think you are ready to use the epinephrine auto injector for treatment?	Yes	19 (18.8)	82 (84.5)	<0.001
	No	31 (30.7)	4 (4.0)	
	I do not know	51 (50.5)	11 (11.5)	
Total (*N*)		101	97	

McNemar’s test used for paired categorical data; *p* < 0.05 considered statistically significant.

## 4 Discussion

Several studies in Saudi Arabia have measured the knowledge of food allergy and anaphylaxis among schoolteachers. These studies revealed a significant lack of basic knowledge about food allergy, recognition of anaphylactic symptoms and signs, and the immediate action required for allergic reactions, including the use of an epinephrine auto-injector (5, 14–17). The recommendations from these studies included providing training programs for schools and educational campaigns. In this pilot exploratory study, we provided an educational simulation intervention and assessed teachers’ knowledge and attitudes toward food allergy and anaphylaxis in primary schools before and after the simulation. The assessment focused on three aspects: identification of anaphylaxis through recognition of signs and symptoms, attitude toward the guidelines and protocols in schools for managing severe allergic reactions, and participants’ readiness to use an epinephrine auto-injector. The findings demonstrated that the simulation intervention was effective in improving participants’ knowledge of the symptoms and signs, especially critical ones such as swelling of the lips, tongue, face, and eyes, as well as shortness of breath (*p* < 0.001), which are key indicators of anaphylaxis. Interestingly, the response categories “None of them” and “I do not know” were both significantly reduced post-intervention (*p* < 0.001), with all respondents moving away from these answers, suggesting again an increased awareness and understanding of the symptoms associated with the condition. A study in Houston, USA, measured the knowledge of school personnel before and after a 1-h educational session on food allergies. The study revealed that the training significantly improved teachers’ knowledge and attitudes toward food allergies, particularly in the early recognition of anaphylaxis and the use of epinephrine auto-injectors, highlighting the importance of educational interventions in enhancing preparedness for allergic reactions in schools (18).

In the current study, there was a statistically significant improvement in self-assessed knowledge scores following the simulation on a 10-point scale, with the mean knowledge score increasing from 4.46 (pre-intervention) to 8.21 (post-intervention) [*p* < 0.001; 95% CI (−5, −3)]. Additionally, a significant gender-based difference was observed in pre-simulation attitudes. However, the simulation appeared to equally benefit both male and female participants, resulting in similar post-intervention outcomes. The attitude toward the guidelines and protocols in schools for managing severe allergic reactions has improved. For example, the agreement on the need for special supervision during mealtimes, rules banning food sharing and the use of nuts, and providing supervision on school buses showed significant shifts toward more substantial support. A previous study evaluating the effect of a single educational session has been successful in enhancing preschool teachers’ self-rated confidence, participant knowledge, and attitude toward anaphylactic emergencies, even after follow-ups of 4 to 12 weeks (19).

Simulation-based education is a well-established teaching method for preparing health workers and medical students to comprehensively understand and manage clinical emergencies (6, 20, 21). To our knowledge, it is uncommon to use a case simulation in educating teachers or the public. A study showed that the in-situ simulation improved team confidence and management of anaphylaxis in the allergy clinic among nurses, allergy-immunology fellows and immunologists (21). A similar study conducted on nurses from different units reported an improvement in the diagnosis and management of anaphylaxis through the detection of signs and symptoms and the rapid use of an epinephrine auto-injector (22).

These results underline the effectiveness of the simulation intervention in enhancing participants’ awareness and proactive attitudes toward anaphylaxis management in educational settings. One of the strengths of this pilot study lies in its use of simulation-based education as a practical and experiential intervention method, and its application in a real-world school setting with teachers who regularly interact with at-risk children. Additionally, the pre–post design and use of validated instruments provide reliable evidence of the short-term educational impact. However, several limitations warrant consideration.

First, although the original sample size was calculated with a 20% attrition buffer, the final post-test sample (*n* = 97) was below the initial projection. To address this, a *post hoc* power analysis was conducted, demonstrating 84% power to detect medium-sized effects, suggesting that the primary outcomes remain statistically robust. Second, while statistical significance was observed across several domains, we also reported effect sizes (e.g., rank-biserial correlation, Cohen’s r) to reflect the practical magnitude of change. This improves interpretability beyond reliance on *p*-values alone. Third, due to the exploratory nature of this pilot study, we did not apply formal corrections for multiple comparisons. This decision was made to preserve statistical sensitivity in a modest sample and avoid inflating Type II error. Nonetheless, we recognize the increased risk of Type I error and recommend cautious interpretation of secondary outcomes. Lastly, the study was conducted in a single region and relied partially on self-reported measures, which may be subject to recall or response biases. Future studies should consider larger, multi-center designs and longitudinal follow-up to assess the durability of simulation training outcomes.

A study conducted in Japan, involving teachers, school nurses, and caregivers working with children who were prescribed epinephrine auto-injectors, demonstrated that practical training significantly increased their confidence and self-efficacy in managing allergic emergencies (23). Many studies from different countries have also identified significant deficiencies in school readiness to treat students with anaphylaxis, including the initiation of management, the availability of epinephrine in schools, and staff training to administer epinephrine (11, 24–26). The inadequate handling of epinephrine auto-injectors in children with anaphylaxis suggests that more effort should be devoted to educating school staff about the proper use of epinephrine, as simulation-based training could be a practical approach to enhance their practice.

This study has some limitations. First, although the original sample size was calculated with a 20% attrition buffer, the final post-test sample (*n* = 97) was slightly below the target. A *post hoc* power analysis, however, confirmed sufficient sensitivity (84% power) to detect medium-sized effects. Given these constraints, the study should be interpreted as a pilot exploratory investigation, providing preliminary evidence that can inform the design of larger, multi-center trials. Third, outcomes relied partly on self-reported measures, which are subject to recall or response bias. Fourth, while no formal corrections for multiple testing were applied due to the exploratory design, we acknowledge the risk of Type I error and recommend cautious interpretation of secondary outcomes. Finally, with only six schools included, clustering effects could not be fully explored. Larger, multi-center studies with longitudinal follow-up are needed to confirm the durability and broader applicability of these findings.

Despite these limitations, the study’s findings were consistent with the existing literature in both school and clinical settings. Moreover, the implemented simulation has increased understanding of anaphylaxis, shifted attitudes positively toward allergy policies, and improved staff readiness to use epinephrine. These findings, though preliminary, suggest the potential of simulation-based training as a scalable tool for school preparedness.

## 5 Recommendations

This study highlights the importance of simulation-based education as a practical and scalable approach to enhancing school preparedness for managing food allergies and anaphylaxis. Based on our findings, we recommend that education authorities integrate regular simulation sessions into teacher training and professional development programs. Schools should establish clear anaphylaxis management protocols, ensure the availability of epinephrine auto-injectors, and designate trained staff to act promptly during emergencies. At the policy level, incorporating allergy preparedness into national school health frameworks would enhance systemic readiness and reduce preventable risks. Future research should focus on evaluating the long-term retention of knowledge and skills, assessing the scalability of simulation-based interventions across diverse educational settings, and conducting cost-effectiveness analyses to guide sustainable implementation.

## 6 Conclusion

In conclusion, the rising prevalence of food allergies among school-aged children underscores the urgent need for effective preparedness strategies. This pilot exploratory study demonstrates that simulation-based education can enhance knowledge, attitudes, and readiness among school staff to manage anaphylaxis. While the findings are promising, they should be interpreted cautiously, and larger multi-center studies are required to establish generalizability and long-term impact. By integrating such training into school health frameworks and reinforcing policies on supervision and food safety, schools can ensure a safer environment for children with allergies.

## Data Availability

The original contributions presented in the study are included in the article/Supplementary material, further inquiries can be directed to the corresponding author/s.
