# Supplementary material for: Evaluating teachers’ knowledge and attitude toward food allergy and anaphylaxis: a pilot simulation-based questionnaire study in Rabigh elementary schools
Source: Front Public Health. 2025 Oct 29;13:1631540. doi: 10.3389/fpubh.2025.1631540 (PMC12605080; doi:10.3389/fpubh.2025.1631540)
Supplement: Supplementary file 1 [file Table_1.DOCX]

**Supplementary materials**

Table S1. Attitude toward the guidelines and protocols in school for the management of severe allergic reactions.

| **Questions** | **Strongly Agree** | **Agree** | **Neutral** | **Disagree** | **Strongly Disagree** |
| --- | --- | --- | --- | --- | --- |
| Preparing and facilitating direct communication with emergency services in case of anaphylaxis. | | | | | |
| Pre | 77(76.2) | 1(17.8) | 5(5.0) | 0(0.0) | 1(1.0) |
| Post | 94(93.0) | 7(7.0) | 0(0.0) | 0(0.0) | 0(0.0) |
| Assigning a specific staff member to directly handle allergic reactions. | | | | | |
| Pre | 72(71.3) | 18(17.8) | 7(6.9) | 2(2.0) | 2(2.0) |
| Post | 79(78.2) | 1(16.8) | 5(5.0) | 0(0.0) | 0(0.0) |
| Defining the role of each teacher or administrator regarding their specific responsibilities in handling emergency cases. | | | | | |
| Pre | 73(72.3) | 22(21.8) | 6(6.0) | 0(0.0) | 0(0.0) |
| Post | 81(80.2) | 15(14.9) | 5(5.0) | 0(0.0) | 0(0.0) |
| Identifying students who have food allergies or anaphylaxis | | | | | |
| Pre | 80(79.2) | 15(14.9) | 3(3.0) | 3(3.0) | 0(0.0) |
| Post | 81(80.2) | 12(11.9) | 6(6.0) | 0(0.0) | 0(0.0) |
| Being prepared to handle a student showing allergy symptoms is necessary when their medical history is unknown. | | | | | |
| Pre | 75(74.3) | 18(17.8) | 4(4.0) | 4(4.0) | 0(0.0) |
| Post | 83(82.2) | 11(10.9) | 7(7.0) | 0(0.0) | 0(0.0) |
| Providing clear guidance for staff handling food to prevent food allergy (e.g., in the cafeteria). | | | | | |
| Pre | 83(82.2) | 15(14.9) | 3(3.0) | 0(0.0) | 0(0.0) |
| Post | 84(83.2) | 11(10.9) | 6(6.0) | 0(0.0) | 0(0.0) |
| Having special supervision during mealtimes for students with food allergies. | | | | | |
| Pre | 64(63.4) | 27(26.7) | 7(6.9) | 3(3.0) | 0(0.0) |
| Post | 75(74.3) | 16(15.8) | 5(5.0) | 5(5.0) | 0(0.0) |
| Having rules that prohibit food sharing among students in schools. | | | | | |
| Pre | 62(61.4) | 26(25.7) | 6(5.9) | 4(4.0) | 3(3.0) |
| Post | 81(80.2) | 13(12.9) | 1(1.0) | 6(6.0) | 0(0.0) |
| Having a rule banning nuts in schools. | | | | | |
| Pre | 59(58.4) | 24(23.8) | 11(10.9) | 7(6.9) | 0(0.0) |
| Post | 81(80.2) | 13(12.9) | 1(1.0) | 2(2.0) | 0(0.0) |
| Providing supervision on school buses for students with food allergies. | | | | | |
| Pre | 59(58.4) | 26(25.7) | 8(7.9) | 7(6.9) | 1(1.0) |
| Post | 77(76.2) | 13(12.9) | 5(5.0) | 1(1.0) | 1(1.0) |
